# Chemogenetic activation of central gastrin‐releasing peptide‐expressing neurons elicits itch‐related scratching behavior in male and female mice

**DOI:** 10.1002/prp2.790

**Published:** 2021-05-17

**Authors:** Norikazu Kiguchi, Yohji Fukazawa, Ayano Saika, Daisuke Uta, Fumihiro Saika, Tomoe Y. Nakamura, Mei‐Chuan Ko, Shiroh Kishioka

**Affiliations:** ^1^ Department of Pharmacology Wakayama Medical University Wakayama City Wakayama Japan; ^2^ Department of Physiological Sciences School of Pharmaceutical Sciences Wakayama Medical University Wakayama City Wakayama Japan; ^3^ Department of Anatomy Kansai University of Health Sciences Sennan‐gun Osaka Japan; ^4^ Department of Applied Pharmacology Faculty of Pharmaceutical Sciences University of Toyama Toyama City Toyama Japan; ^5^ Department of Physiology and Pharmacology Wake Forest University School of Medicine Winston‐Salem NC USA; ^6^ Faculty of Wakayama Health Care Sciences Takarazuka University of Medical and Health Care Wakayama City Wakayama Japan

**Keywords:** dorsal horn, DREADD, GRP, GRPR, pruritus, sex, spinal cord

## Abstract

Several lines of evidence have clarified that the key transmission pathways of itching sensation travel from the periphery to the central nervous system (CNS). Despite the functional significance of gastrin‐releasing peptide (GRP) and its cognate receptor in the itch processing mechanism in the spinal dorsal horn (SDH), the roles of GRP‐expressing (GRP^+^) neurons in different regions remain unclear. This study aimed to determine whether GRP^+^ neurons in the CNS directly modulated itch processing. To specifically activate spinal and supraspinal GRP neurons by the designer receptors exclusively activated by designer drugs (DREADDs) system, CAG‐LSL‐Gq‐DREADD mice were crossed with GRP‐Cre mice, resulting in the development of GRP‐hM3Dq mice. Immunohistochemistry showed that hM3Dq was highly expressed in the SDH and brainstem closely related to sensory processing. The intraperitoneal, intrathecal, or intracerebroventricular administration of clozapine‐N‐oxide, an agonist of hM3Dq, strongly elicited dermatome‐dependent itch‐related scratching behavior, but did not change pain sensitivity. Importantly, GRP‐Gq‐DREADD‐mediated scratching behavior in GRP‐hM3Dq mice was not affected by the ablation of transient receptor potential vanilloid 1^+^ sensory C‐fibers, and it was also observed to a similar degree under chronic itch conditions. Furthermore, there were no significant sex differences in the scratching behavior elicited by GRP‐Gq‐DREADD, suggesting that itch‐dominant roles of central GRP^+^ neurons might be common in both sexes, at least under normal physiological conditions. These novel findings not only contribute to understanding the functional roles of central GRP^+^ neurons further, but also propose the development of future effective therapeutics for intractable itching.

## Graphical abstract

Chemogenetic activation of spinal GRP+ neurons by CNO administration in GRP‐hM3Dq mice elicited robust dermatome‐dependent scratching behavior in male and female mice, suggesting that itch‐processing roles of GRP+ neurons might be common in both sexes under physiological condition.

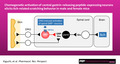

**Abbreviations**
AMPAR: α‐amino‐3‐hydroxy‐5‐methyl‐4‐isoxazolepropionic acid receptor
CNO: clozapine‐N‐oxide
CNS: central nervous system
DCP: diphenylcyclopropenone
DREADD: designer receptors exclusively activated by designer drugs
DRG: dorsal root ganglia
GRP: gastrin‐releasing peptide
GRPR: GRP receptor
HA: hemagglutinin
PBS: phosphate‐buffered saline
RTX: resiniferatoxin
SDH: spinal dorsal horn
Tg: transgenic
TRPV1: transient receptor potential vanilloid 1

## 1 INTRODUCTION

Nociceptive somatosensory systems are indispensable as a warning system to detect abnormalities or disorders and avoid excessive damage to organs. An itch is an uncomfortable sensation that evokes a desire or reflex to scratch, and chronic itching may be associated with several skin diseases (e.g., atopic dermatitis, contact dermatitis, and psoriasis), which severely affects the quality of life.^1^, ^2^ To date, several lines of evidence have clarified that the key transmission pathways of itching sensation travel from the periphery to the central nervous system (CNS).^3^, ^4^, ^5^ In the skin, many pruritogens activate cognate pruriceptors expressed on the primary afferent nociceptive sensory neurons.^6^, ^7^ Particularly, transient receptor potential vanilloid 1 (TRPV1) and Mas‐related G‐protein‐coupled receptor A3‐expressing C‐fibers are known to be crucial in the transmission of itch‐related information to the spinal dorsal horn (SDH).^8^, ^9^

Among a variety of neuropeptides in the SDH, the gastrin‐releasing peptide (GRP) plays a fundamental role in the itch processing mechanism.^10^, ^11^, ^12^ Intrathecal (i.t.) administration of GRP strongly elicited itch‐related scratching behavior in both rodents and primates.^13^, ^14^ Moreover, there are several reports showing that inhibition of GRP receptor (GRPR)‐expressing (GRPR^+^) itch‐responsive neurons in the SDH attenuates both acute and chronic itching.^15^, ^16^, ^17^ However, despite the functional significance of the GRP‐GRPR system in itch processing, the distribution of endogenous GRP remains controversial. Some reports demonstrated that GRP was expressed in the C‐fibers of the dorsal root ganglia (DRG) neurons,^18^, ^19^ while others emphasized that GRP was released from excitatory interneurons in the SDH.^20^, ^21^, ^22^ Using transgenic (Tg) mice, GRP promoter‐driven fluorescent markers were expressed in the SDH,^20^, ^21^ indicating that functional GRP^+^ neurons might be located in the SDH. These findings are also supported by single‐cell RNA sequencing analysis that revealed that GRP mRNA was detected in the glutamatergic interneurons of the SDH.^22^ In contrast, Barry et al. determined that GRP was expressed in the DRG neurons using newly developed GRP‐knock‐in mice.^23^ They found that pruritogens induced Ca^2+^ responses in GRP^+^ sensory neurons and that optogenetic activation of GRP^+^ sensory neurons elicited itch‐related scratching behavior. Furthermore, ablation of the GRP^+^ neurons in the DRG, but not the SDH, affected acute itching, suggesting that spinal GRP^+^ neurons might be dispensable in the itching sensation.^23^ Although there is a consistent understanding that expression levels of GRP in the SDH are significantly greater than those in the DRG, localization of functionally important GRP^+^ neurons for itch processing remains controversial.

This study aimed to determine whether GRP^+^ neurons in the CNS directly affected itch processing. In our previously published study, since GRP‐Cre (Tg) mice predominantly reflected GRP expression in the CNS,^15^ but not in the sensory neurons, the GRP‐Cre‐dependent designer receptors exclusively activated by designer drugs (DREADDs) system can explore the roles of GRP^+^ neurons in the CNS. Herein, we investigated whether chemogenetic activation of spinal and supraspinal GRP^+^ neurons by Gq‐DREADD elicited itching sensation under normal and pathological conditions. We confirmed that central GRP^+^ neurons have the ability to evoke itching, regardless of the expression of GRP in the sensory neurons.

## 2 MATERIALS AND METHODS

### 2.1 Mice

All animal experiments were approved by the Animal Research Committee of Wakayama Medical University and were carried out in accordance with the in‐house guidelines for the care and use of laboratory animals of Wakayama Medical University and the ARRIVE guidelines. Male and female C57BL/6J mice (8–12 weeks old) were used in all experiments. CAG‐LSL‐Gq‐DREADD mice (B6N;129‐Tg[CAG‐CHRM3*,‐mCitrine]1Ute/J; stock #026220) and GRP‐Cre (Tg) mice (B6.FVB(Cg)‐Tg[Grp‐cre]KH288Gsat/Mmucd; stock #037585) were purchased from The Jackson Laboratory and Mutant Mouse Resource & Research Centers and were maintained as heterozygous genotypes. Both strains were crossed for Cre‐dependent expression of the Gq‐DREADD system in GRP‐expressing cells. Mice were housed in plastic cages in a temperature‐controlled room (23–24℃, 60%–70% humidity) with a 12‐h dark/light cycle and provided with water and food ad libitum.

### 2.2 Drug administration

Clozapine‐N‐oxide (CNO; Enzo Life Sciences) was dissolved in sterile phosphate‐buffered saline (PBS) and diluted as needed. To observe the systemic effects of GRP‐Gq‐DREADD, intraperitoneal (i.p.) injection of CNO (0.1 ml/10 g body weight) was administered in conscious mice. To examine the central effects of GRP‐Gq‐DREADD, CNO (5 µl) was administered either by i.t. or intracerebroventricular (i.c.v.) routes in isoflurane‐anesthetized mice. The i.t. injection was administered in the region between the spinal L5 and L6 vertebrae using a 30‐gauge needle fitted with a Hamilton microsyringe.^24^ The i.c.v. injection was administered into the left lateral ventricle using a 26‐gauge needle fitted with a Hamilton microsyringe, as described previously.^25^ Chloroquine diphosphate (Sigma‐Aldrich) was administered intradermally (i.d.) in the nape of isoflurane‐anesthetized mice. The drugs were administered in a volume of 100 µl using a 30‐gauge needle fitted to a 1‐ml syringe after shaving the fur at the injection site. Resiniferatoxin (RTX, Cayman Chemical), a natural capsaicin analog, was dissolved in dimethyl sulfoxide and diluted with sterile saline. To ablate TRPV1^+^ sensory neurons, increasing doses (30, 70, and 100 μg/kg) were administered subcutaneously (s.c.) for 3 consecutive days.^26^

### 2.3 Contact dermatitis

Diphenylcyclopropenone (DCP; Wako) was dissolved in acetone. For sensitization, 0.2 ml of 2% DCP was used after shaving the fur of the back of isoflurane‐anesthetized mice.^15^ Seven days after sensitization, the mice were challenged with 0.2 ml of 1% DCP once a day for 7 consecutive days. The number of scratching bouts was measured for 40 min immediately after i.p. CNO administration.

### 2.4 Scratching behaviors

To evaluate the degree of itching sensation in rodents, the frequency of scratching behaviors using hind paw is generally measured in basic studies. Mice were habituated for 60 min in plastic cages (20 × 12 × 12 cm^3^) with a small amount of bedding. After the administration of CNO or chloroquine, scratching behavior was videotaped for 60 min with video camera. The number of scratching bouts was measured at 10‐min intervals for 30 min (chloroquine) or 40 min (CNO) by playing back the videotape as reported previously.^27^ One scratching bout was defined as lifting the hind paw to scratch the trunk area of the body, face/head, or nape area following i.t. or i.d. injection and then returning the paw to the floor or to the mouth for licking. All analyses were performed in a blinded fashion.

### 2.5 Immunohistochemistry

The lumbar (L4–L5) spinal cord or whole brain was harvested from the euthanized mice after transcardiac perfusion in PBS and fixed in 4% paraformaldehyde. The specimens were then post‐fixed in 4% paraformaldehyde and cryoprotected in 30% sucrose at 4°C overnight. After embedding in a freezing compound (Sakura), the frozen spinal cord was cut longitudinally into 30‐µm‐thick sections with a cryostat and allowed to float in PBS, whereas the brain was cut coronally into 14‐µm‐thick sections and mounted on glass slides. The sections were treated with PBS containing 0.3% Triton X‐100 (PBST) for 1 h and then blocked with 5% normal donkey serum in 0.3% PBST at 15–25°C for 2 h. The sections were incubated with primary antibodies against hemagglutinin (HA) epitope‐tag (mouse monoclonal, 1:250; BioLegend) and c‐fos (rabbit polyclonal, 1:50; Santa Cruz Biotechnology) at 4°C overnight. All antibodies were diluted in 1% normal donkey serum in 0.1% PBST. Subsequently, sections were rinsed in PBST and incubated with Alexa 488‐conjugated secondary antibodies (1:300; Abcam) at 15–25°C for 2 h. Sections were rinsed in PBST and then incubated with Hoechst 33342 (Thermo Fisher Scientific) at 15–25℃ for 10 min. Finally, the sections were coverslipped with a mounting medium on a glass slide. Fluorescence images of SDH were detected using a confocal laser scanning microscope (Carl Zeiss). For quantification of c‐fos in each mouse, the number of positive cells in the cervical SDH was calculated as the average of three randomly selected sections from one segment of each mouse before c‐fos staining was viewed. All images of c‐fos labeling were captured simultaneously with the same camera settings, and the persons performing the counts were blinded to the treatment groups. The brightness and contrast of fluorescent micrographs were minimally processed and colorized as needed using Adobe Photoshop as previously described.^15^

### 2.6 Paw‐withdrawal tests

To evaluate thermal sensitivity, the withdrawal threshold was determined using the Hargreaves test.^28^ Mice were placed on a glass sheet and covered with a clear acrylic box. After adaptation for 2–3 h, a radiant heat source (IITC 390 Plantar Test Analgesia Meter, Neuroscience) was positioned under the glass sheet and applied to the plantar surface of the hind paw. Withdrawal latencies were evaluated based on the mean latency of three stimulations. A cutoff latency of 20 s was set to avoid tissue damage. To evaluate mechanical sensitivity, the 50% withdrawal threshold was determined using the von Frey test.^29^ Mice were placed on a 5 × 5‐mm wire mesh grid floor and covered with a clear acrylic box. After adaptation for 2–3 h, calibrated von Frey filaments (Neuroscience) were applied to the middle of the plantar surface of the hind paw through the bottom of the mesh floor. In the paradigm of the up‐down method, testing was initiated with a 0.4‐g force in the middle of the series (0.02, 0.04, 0.07, 0.16, 0.4, 0.6, 1.0, 1.4, and 2.0 g). Stimuli were always presented in consecutive fashion, either ascending or descending. In the absence of a paw withdrawal response to the selected force, a stronger stimulus was applied. In the presence of paw withdrawal, the next weaker stimulus was chosen. Based on the responses to the series of the von Frey filament, the 50% paw withdrawal threshold was calculated.

### 2.7 Data analysis

Data were presented as mean ± standard error of the mean. Statistical analyses were performed using Student's *t*‐test and one‐way analysis of variance, followed by Tukey's multiple comparison test. The result of each analysis was shown in the text, and statistical significance was set at *p*‐value <.05.

### 2.8 Nomenclature of targets and ligands

Key protein targets and ligands in this article are hyperlinked to corresponding entries in http://www.guidetopharmacology.org, the common portal for data from the IUPHAR/BPS Guide to PHARMACOLOGY,^30^ and are permanently archived in the Concise Guide to PHARMACOLOGY 2019/20.^31^

## 3 RESULTS

### 3.1 Effects of systemic GRP‐Gq‐DREADD on itch processing

**FIGURE 1:**
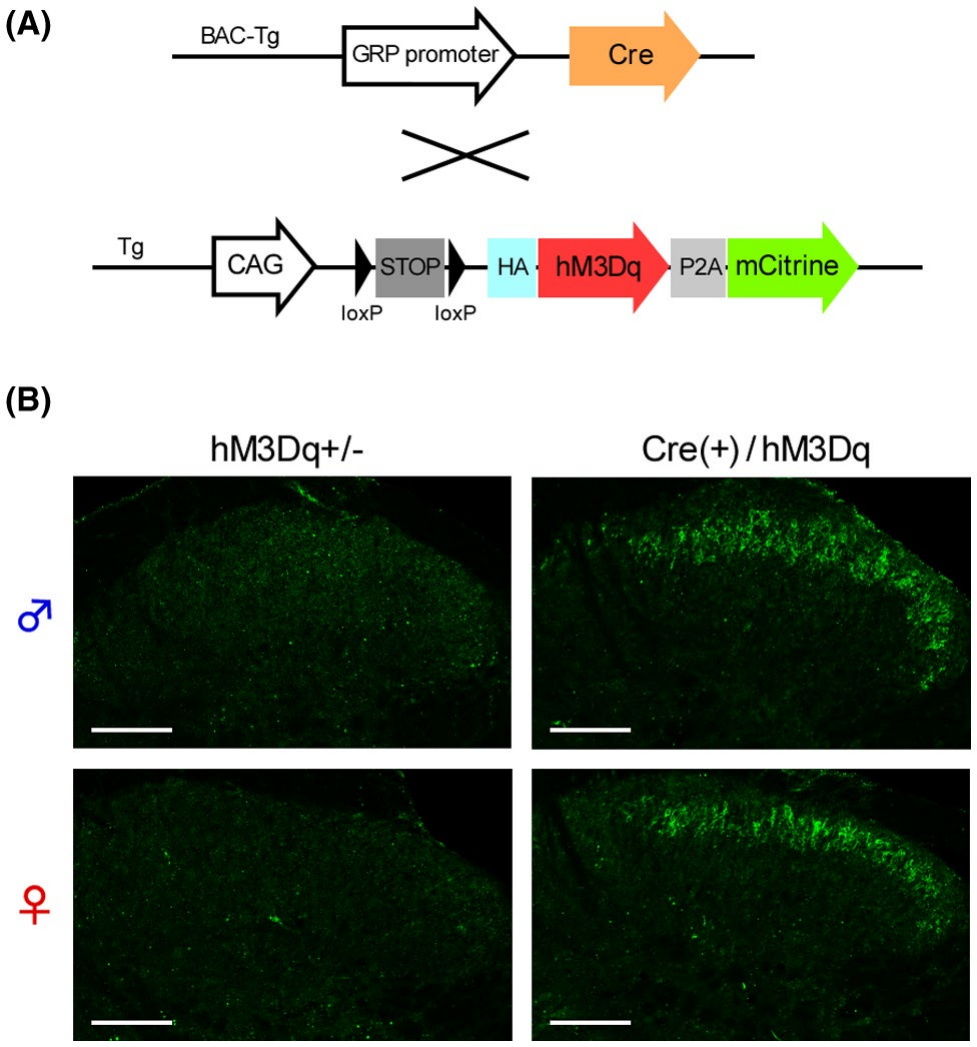
Expression of gastrin‐releasing peptide‐Cre‐driven Gq‐designer receptors exclusively activated by designer drugs system in the spinal dorsal horn. (A) CAG‐LSL‐Gq‐DREADD mice were crossed with GRP‐Cre mice to develop GRP‐hM3Dq mice; (B) The Cre‐dependent expression of HA‐tagged hM3Dq in the SDH of GRP‐hM3Dq mice, but not of hM3Dq heterozygous (control) mice, in both sexes was visualized using immunohistochemistry (Scale bars = 100 µm). DREADD, designer receptors exclusively activated by designer drugs; GRP, gastrin‐releasing peptide; HA, hemagglutinin; SDH, spinal dorsal horn

**FIGURE 2:**
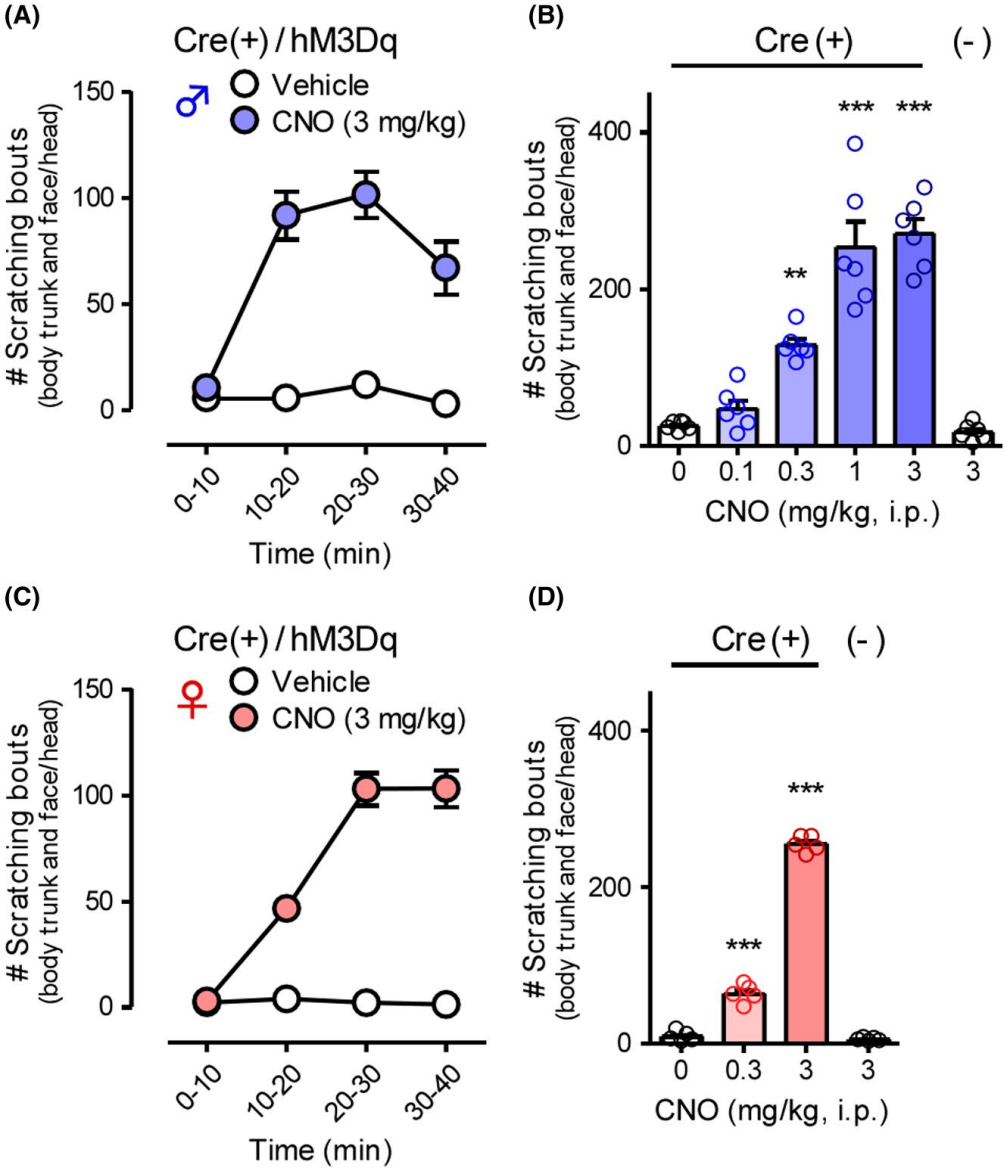
Effects of systemic gastrin‐releasing peptide‐Gq‐designer receptors exclusively activated by designer drugs on itch processing. CNO (0.1–3 mg/kg) was i.p. administered in GRP‐hM3Dq or control mice of both sexes (male: A and B; female: C and D), and scratching bouts in the body trunk and face/head were observed immediately after CNO administration for 40 min. Time course in 10‐min intervals (A and C) and the total number of scratching bouts for 40 min (B and D) are shown. ***p < .001, **p < .01 versus vehicle; each value represents the mean ± SEM (*n* = 5–6). CNO, clozapine‐N‐oxide; GRP, gastrin‐releasing peptide

**FIGURE 3:**
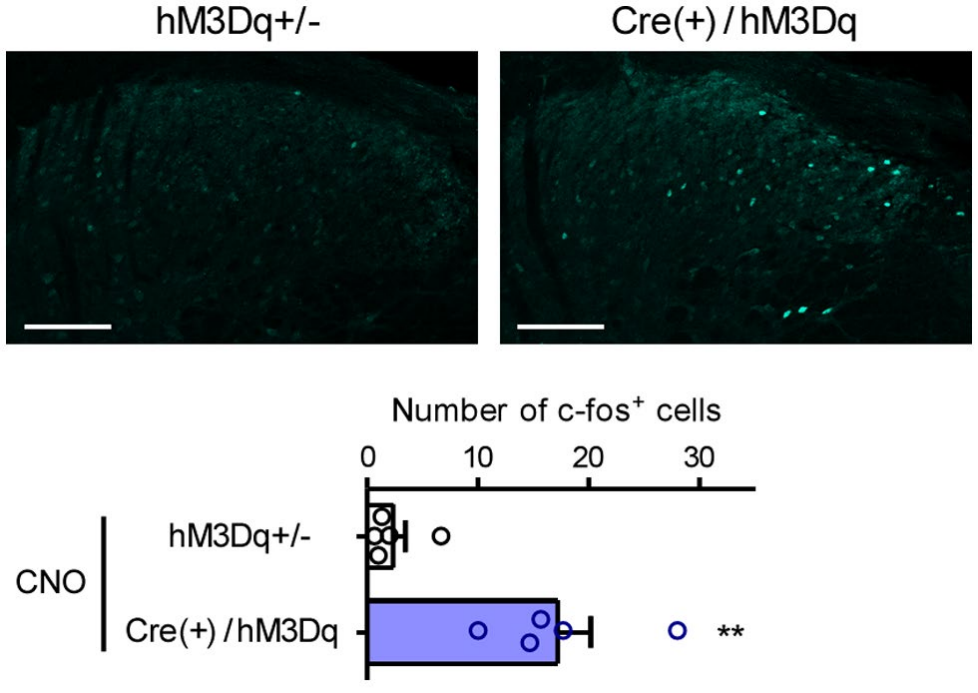
Expression of c‐fos in the spinal dorsal horn by systemic gastrin‐releasing peptide‐Gq‐designer receptors exclusively activated by designer drugs. CNO (1 mg/kg) was i.p. administered in male GRP‐hM3Dq and control mice, and the protein expression of c‐fos in the cervical SDH, 2 h after CNO administration, was visualized using immunohistochemistry (Scale bars = 100 µm). Representative micrographs and mean number of c‐fos^+^ cells are shown. **p < .01 versus control; each value represents the mean ± SEM (*n* = 5). CNO, clozapine‐N‐oxide; GRP, gastrin‐releasing peptide; SDH, spinal dorsal horn

To reveal the functional roles of GRP^+^ neurons in itch processing, we evaluated the effects of chemogenetic activation of such neurons using the GRP‐Cre‐driven Gq‐DREADD system. HA‐tag, indicating hM3Dq expression, was highly expressed in the SDH in the CAG‐LSL‐Gq‐DREADD mice and GRP‐Cre crossed mice (Figure 1A) as compared to the control (hM3Dq heterozygous) mice. There was no difference in the expression patterns of HA‐tag located on cell bodies and neurites in the SDH between the male and female mice (Figure 1B). In male GRP‐hM3Dq mice, systemic i.p. administration of CNO significantly elicited itch‐related scratching behavior in the trunk and face/head regions, and the degree of scratching behavior increased in a dose‐dependent manner (CNO, 0.1–3 mg/kg) (*F*(5,30) = 48.23, *p* < .0001; Tukey's test, *p* < .001). In contrast, CNO (3 mg/kg, i.p.) did not elicit scratching behavior in the control mice (Figure 2A and B). Moreover, i.p. administration of CNO also elicited scratching behavior in the whole body in female mice (*F*(5,24) = 187.9, *p* < .0001; Tukey's test, *p* < .001) (Figure 2C and D), which was consistent with similar expression patterns of hM3Dq in the SDH in the mice of both sexes. To confirm the induction of itching sensation at the spinal level, the expression of c‐fos, an indicator of neural excitation, was evaluated. In GRP‐hM3Dq mice, the number of c‐fos^+^ cells increased in the SDH after i.p. administration of CNO (1 mg/kg) as compared to the control mice (*t*(8) = 4.679, *p* = .0016) (Figure 3).

### 3.2 Effects of region‐selective GRP‐Gq‐DREADD on itch processing

**FIGURE 4:**
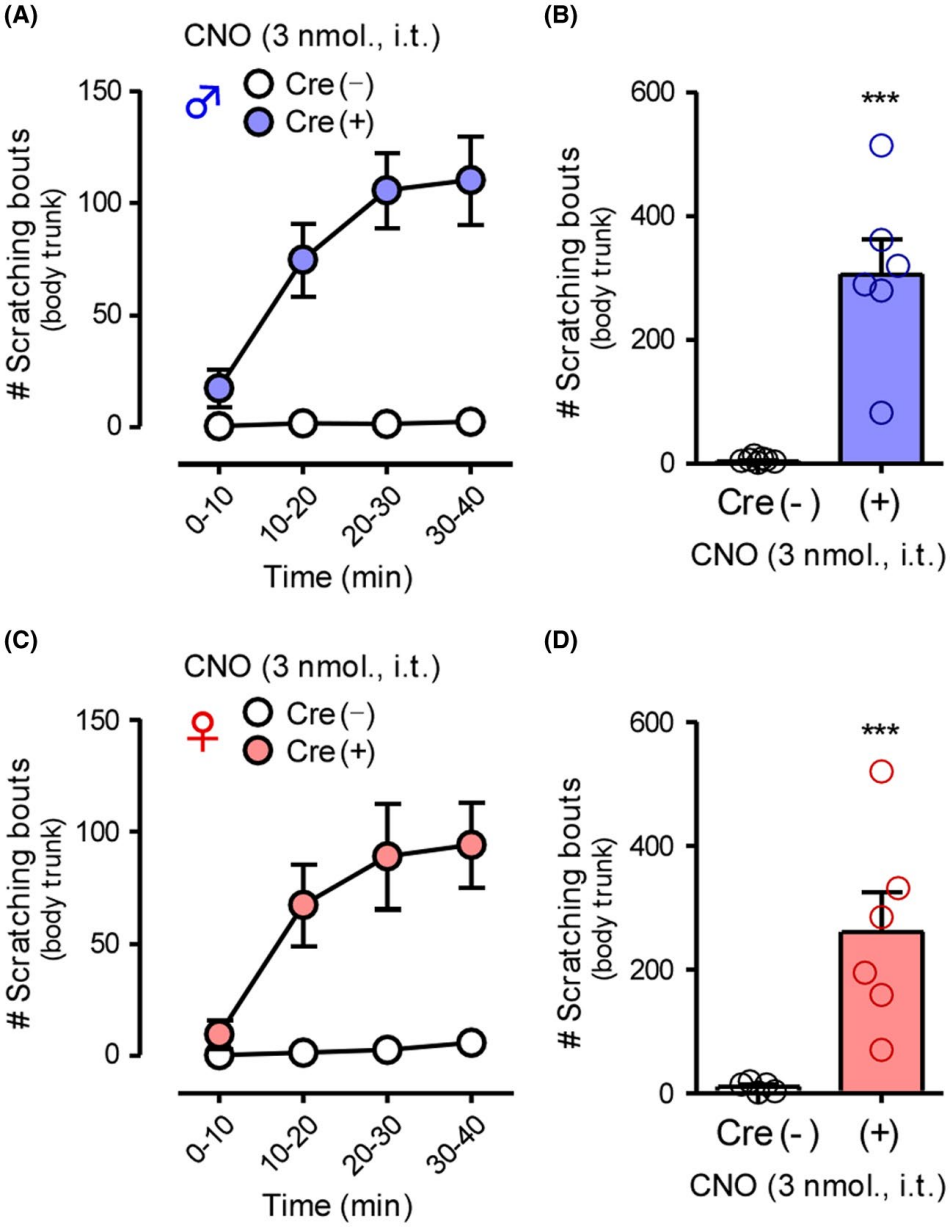
Effects of gastrin‐releasing peptide‐Gq‐designer receptors exclusively activated by designer drugs in the spinal dorsal horn on itch processing. CNO (3 nmol) was i.t. administered in GRP‐hM3Dq and control mice of both sexes (male: A and B; female: C and D), and scratching bouts in the body trunk were observed immediately after CNO administration for 40 min. Time course in 10‐min intervals (A and C) and total number of scratching bouts for 40 min (B and D) are shown. ***p < .001 versus vehicle; each value represents the mean ± SEM (*n* = 5–7). CNO, clozapine‐N‐oxide; GRP, gastrin‐releasing peptide

**FIGURE 5:**
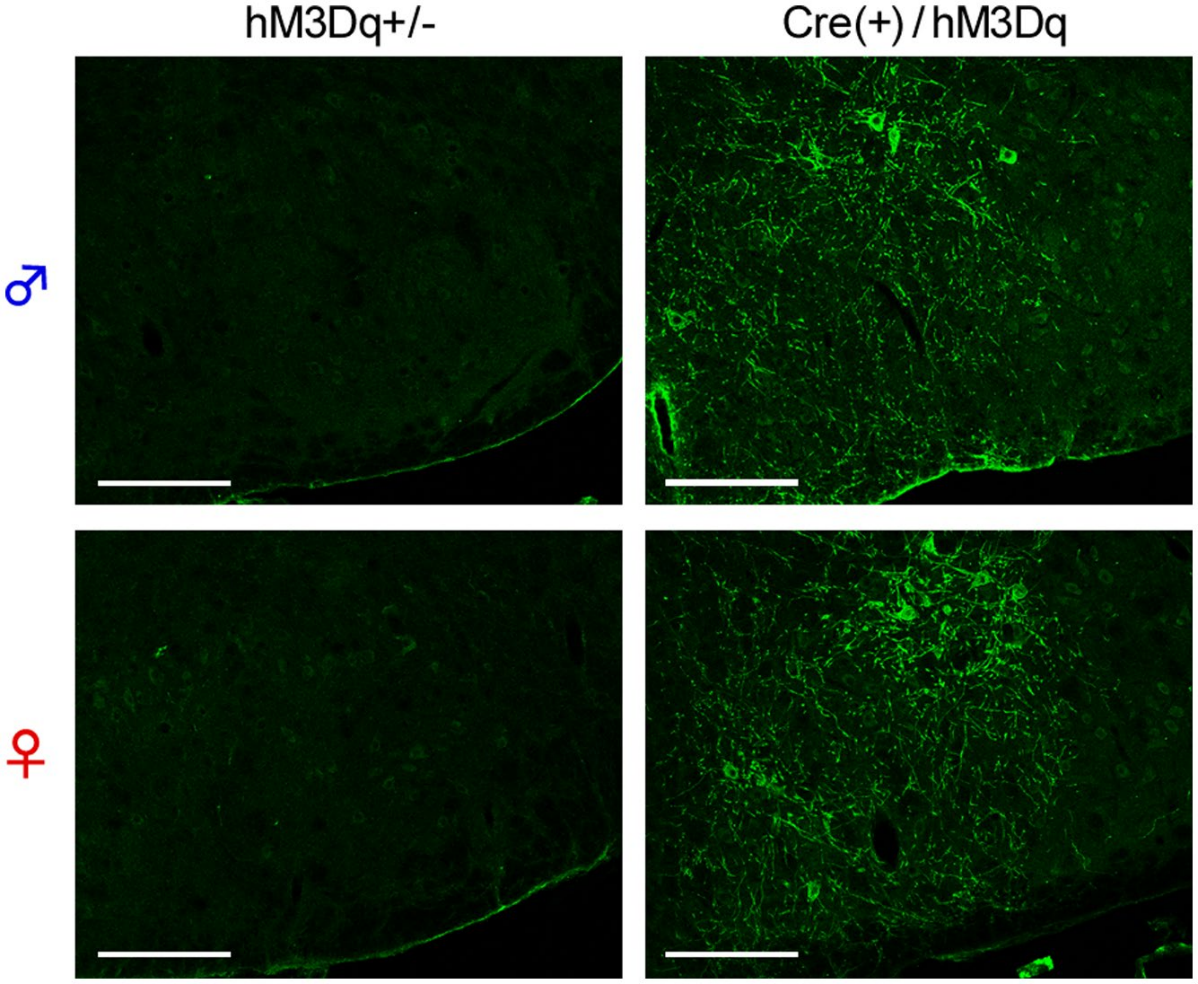
Expression of gastrin‐releasing peptide‐Cre‐driven Gq‐designer receptors exclusively activated by designer drugs system in the facial nucleus. The Cre‐dependent expression of HA‐tagged hM3Dq in the facial nucleus of GRP‐hM3Dq mice of both sexes, but not of hM3Dq heterozygous (control) mice, was visualized using immunohistochemistry (Scale bars = 200 µm). GRP, gastrin‐releasing peptide; HA, hemagglutinin

**FIGURE 6:**
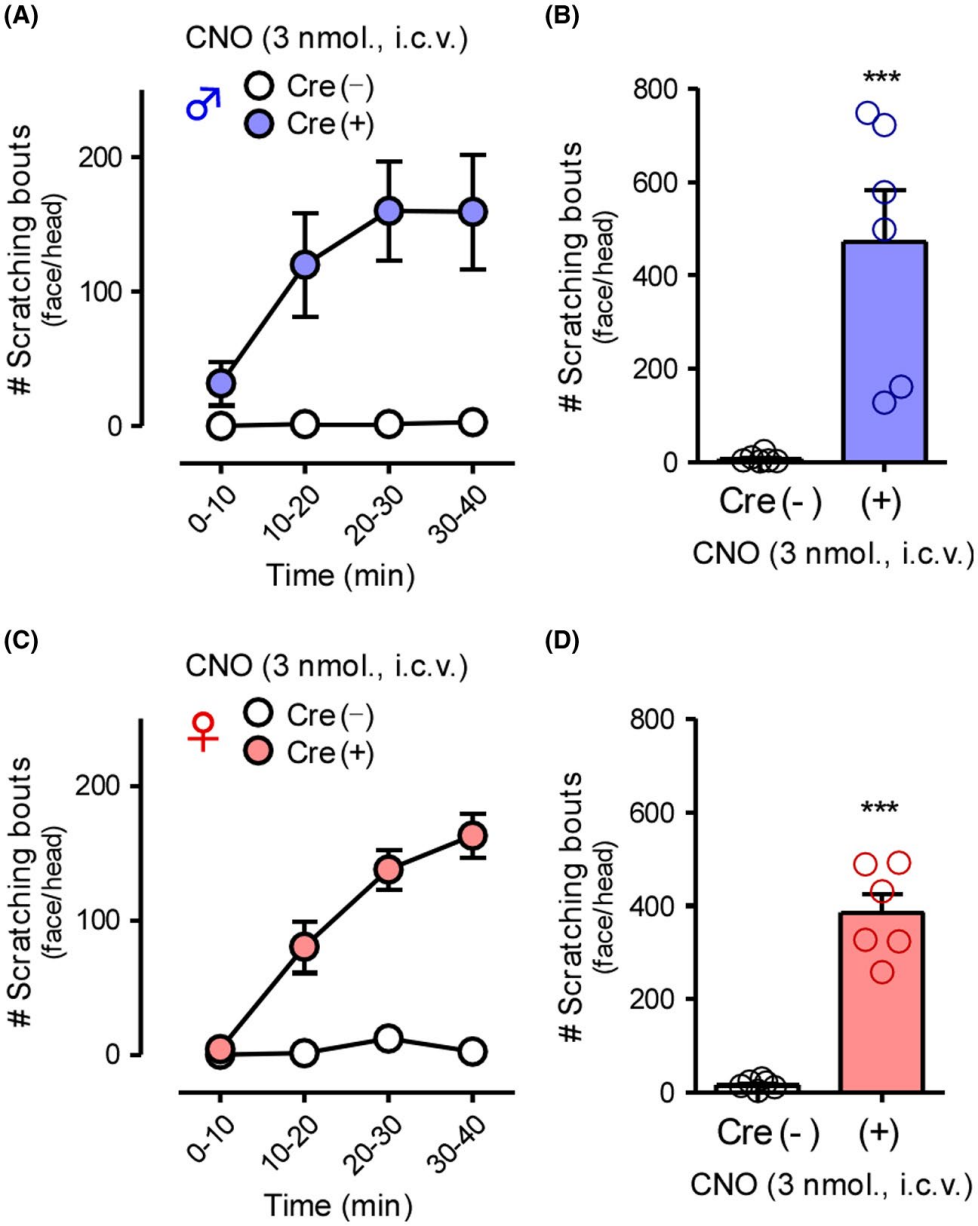
Effects of gastrin‐releasing peptide‐Gq‐designer receptors exclusively activated by designer drugs in the brain on itch processing. CNO (3 nmol) was i.c.v. administered in GRP‐hM3Dq and control mice of both sexes (male: A and B; female: C and D), and the scratching bouts in the face/head were observed immediately after CNO administration for 40 min. Time course in 10‐min intervals (A and C) and total number of scratching bouts for 40 min (B and D) are shown. ***p < .001 versus vehicle; each value represents the mean ± SEM (*n* = 6). CNO, clozapine‐N‐oxide; GRP, gastrin‐releasing peptide

To demonstrate the effects of CNS‐selective activation of GRP^+^ neurons, i.t. CNO was administered in GRP‐hM3Dq mice. Based on our previous report,^15^ 3 nmol of CNO was chosen to observe reasonable effects without any adverse effects. In male and female GRP‐hM3Dq mice, i.t. administration of CNO (3 nmol) elicited robust scratching behavior in the body trunk in a time‐dependent manner, without any sex differences, when compared with the control mice (male: *t*(11) = 5.752, *p* = .0001; female: *t*(9) = 3.509, *p* = .0066) (Figure 4A–D). Next, we investigated the expression of HA‐hM3Dq in the brainstem to determine the roles of GRP^+^ neurons in the trigeminal innervation area. In both male and female GRP‐hM3Dq mice, HA‐tag was highly expressed in the facial nucleus in the different brain regions associated with itching sensation on the face/head (Figure 5). On the other hand, weak expression of HA‐hM3Dq in the spinal trigeminal nucleus‐oral part (Figure S1) was observed in the brainstem of GRP‐hM3Dq mice, but not in the spinal trigeminal nucleus‐caudal part and principal sensory trigeminal nucleus. In male and female GRP‐hM3Dq mice, i.c.v. administration of CNO (3 nmol) elicited robust scratching behavior in the face/head in a time‐dependent manner as compared to the control mice (male: *t*(10) = 4.209, *p* = .0018; female: *t*(10) = 9.242, *p* < .0001). Consistent with the results of i.t. injection administration, there were no sex differences in the i.c.v. CNO‐induced scratching behavior (Figure 6A–D).

### 3.3 Characterization of the itch mediated by GRP‐Gq‐DREADD

**FIGURE 7:**
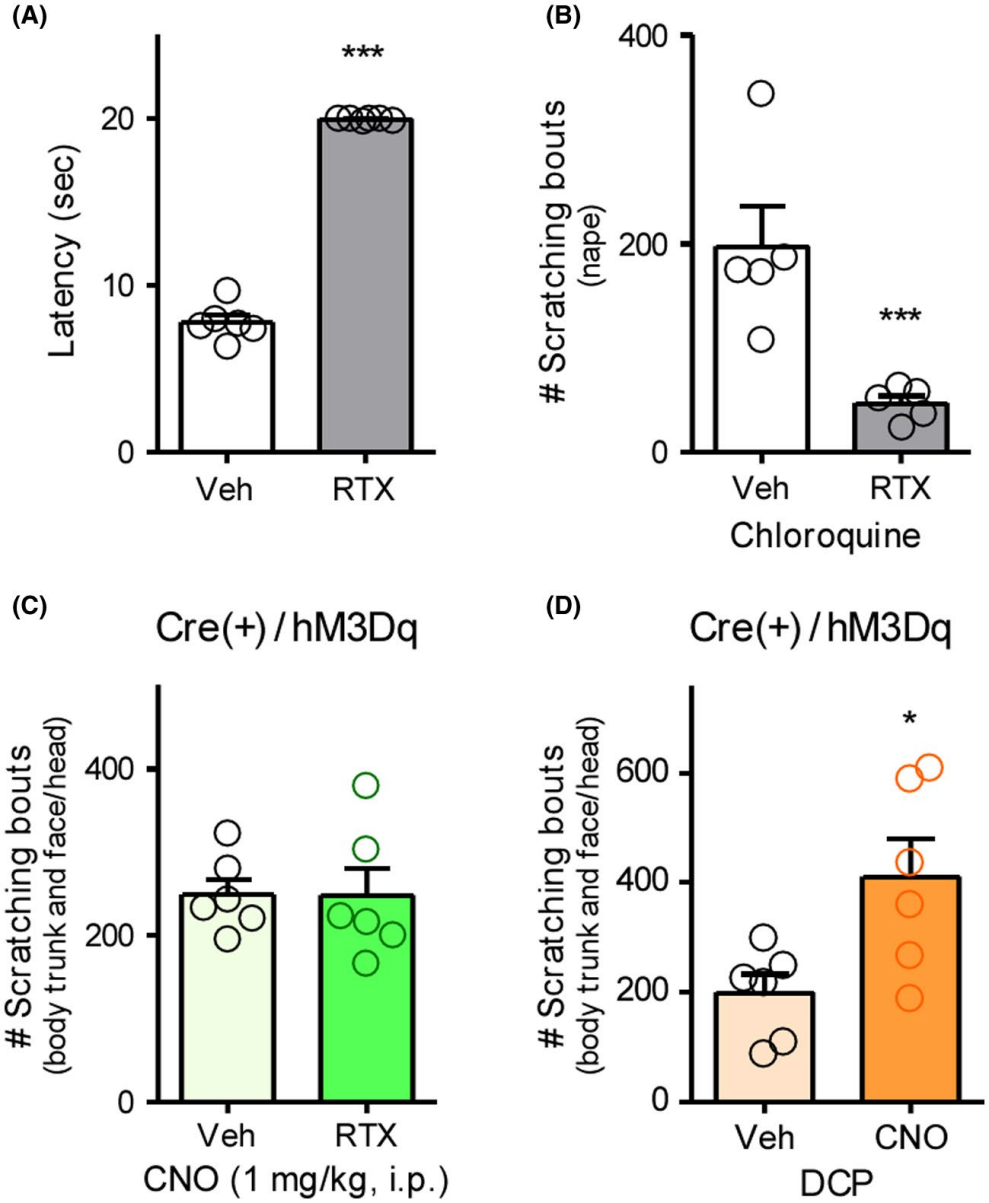
Characterization of scratching behavior elicited by gastrin‐releasing peptide‐Gq‐designer receptors exclusively activated by designer drugs. RTX (30, 70, and 100 µg/kg) was s.c. administered in wild type (A and B) or GRP‐hM3Dq mice (C) for 3 consecutive days, and the mice were kept for 3 weeks. (A) Withdrawal latency to thermal stimuli was assessed by Hargreaves test. (B) Chloroquine (300 nmol) was i.d. administered in the nape of mice, and scratching bouts were observed immediately after chloroquine administration for 30 min. (D) Seven days after sensitization with 2% DCP, 1% DCP was repeatedly applied to GRP‐hM3Dq mice for 7 consecutive days. (C and D) CNO (1 mg/kg) was i.p. administered in mice, and the scratching bouts were observed immediately after CNO administration for 40 min. The total number of scratching bouts is shown (B–D). ***p < .001, *p < .05 versus vehicle; each value represents the mean ± SEM (*n* = 6). CNO, clozapine‐N‐oxide; DCP, diphenylcyclopropenone; GRP, gastrin‐releasing peptide; RTX, resiniferatoxin

To characterize the scratching behaviors caused by chemogenetic activation of central GRP^+^ neurons, itch‐processing TRPV1^+^ sensory neurons were ablated by treatment with RTX. In wild‐type mice, at 3 weeks after RTX (30, 70, and 100 µg/kg, s.c.) treatment, withdrawal latency to heat stimuli was clearly prolonged (*t*(10) = 27.39, *p* < .0001), suggesting the successful ablation of nociceptive TRPV1^+^ C‐fibers (Figure 7A). Moreover, scratching behavior elicited with i.d. administration of chloroquine, a pruritogen‐acting Mas‐related G‐protein‐coupled receptor A3 on C‐fibers, was significantly attenuated in RTX‐treated mice as compared to the vehicle‐treated mice (*t*(8) = 3.775, *p* = .0054) (Figure 7B). Notably, the scratching behavior after i.p. administration of CNO (1 mg/kg) in GRP‐hM3Dq mice was not attenuated by RTX treatment, indicating that chemogenetic activation of GRP^+^ neurons in the CNS can induce a central itching sensation regardless of the excitation of TRPV1^+^ sensory neurons (Figure 7C). Under contact dermatitis induced by repeated application of DCP in GRP‐hM3Dq mice, spontaneous scratching behavior was observed as previously shown.^15^ Even under chronic itching conditions, i.p. administration of CNO (1 mg/kg) significantly enhanced the degree of scratching behavior (*t*(10) = 2.727, *p* = .0213) (Figure 7D).

### 3.4 No effects of GRP‐Gq‐DREADD on pain processing

**FIGURE 8:**
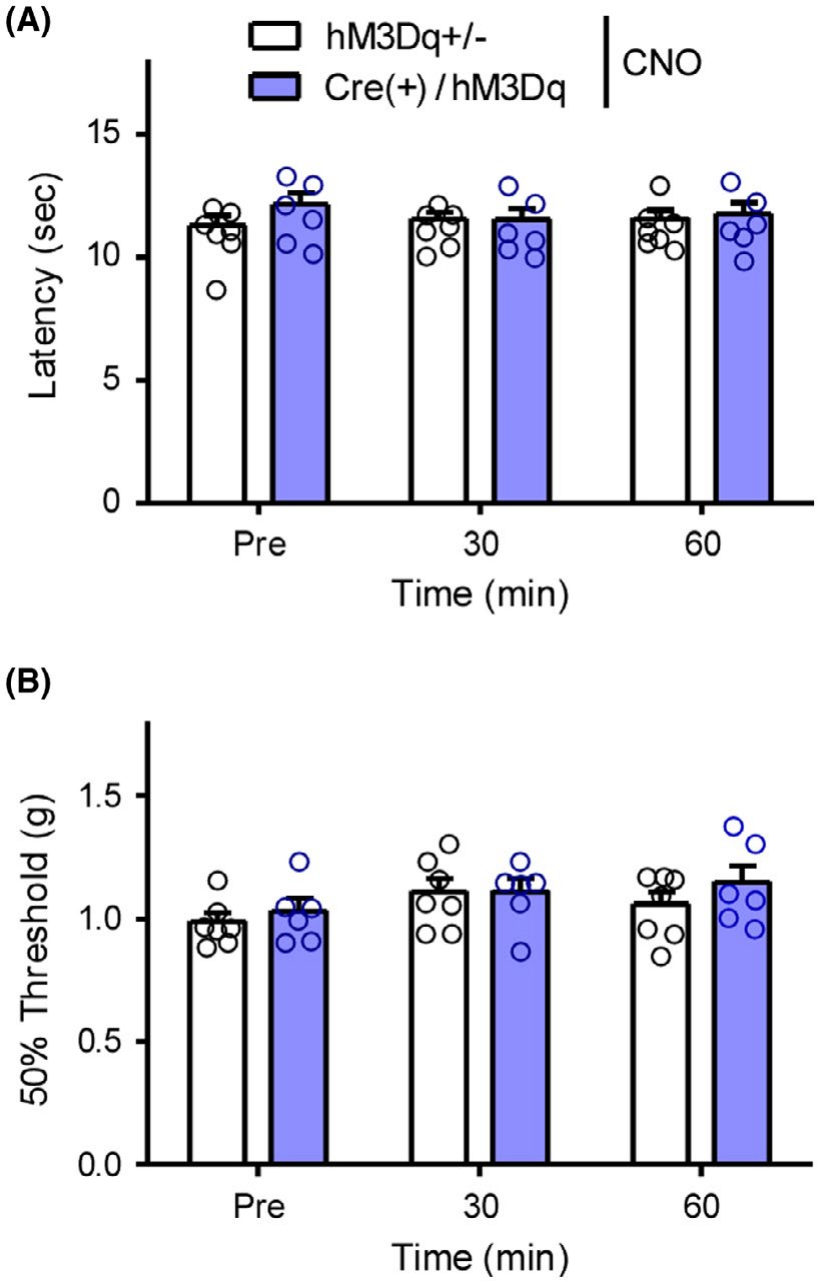
Effects of gastrin‐releasing peptide‐Gq‐designer receptors exclusively activated by designer drugs on pain processing CNO (1 mg/kg) was i.p. administered in male GRP‐hM3Dq and control mice. Withdrawal latency to thermal stimuli and withdrawal threshold to mechanical stimuli were assessed by Hargreaves test (A) and von Frey test (B), respectively. Each value represents the mean ± SEM (*n* = 6–7). CNO, clozapine‐N‐oxide; GRP, gastrin‐releasing peptide

Finally, we determined whether activation of central GRP^+^ neurons affected the thermal and mechanical pain threshold because of the possible relationship between itching and pain. The i.p. administration of CNO (1 mg/kg) did not affect the withdrawal latency to heat stimuli or withdrawal threshold to mechanical stimuli at any time point after administration, suggesting that central GRP^+^ neurons might be dominant in itching sensation (Figure 8).

## 4 DISCUSSION

Originally, Sun et al. suggested that functionally important GRP was produced by sensory C‐fibers and released into the SDH, which integrated the itching sensation.^19^ Later, they also found that GRPR^+^ neurons in the SDH played an essential role in chemical itching by ablation of GRPR^+^ neurons using GRPR‐targeting toxin.^16^ So far, the functional significance of GRPR^+^ neurons has been widely accepted in the scientific community,^3^, ^4^, ^5^, ^11^ though there are several controversial reports regarding the source of itch‐related GRP. Using mice expressing an enhanced green fluorescent protein under the control of the GRP promoter, the protein was clearly detected in the cell body of the GRP neurons in the SDH.^20^, ^21^ Consistent with these findings, Häring et al. reported critical evidence indicating that GRP expression was restricted in the glutamatergic excitatory interneurons in the SDH, but not inhibitory interneurons, using single‐cell RNA sequencing analysis.^22^ In contrast, Barry et al. recently emphasized that functional GRP^+^ neurons are primary sensory neurons regardless of the lower expression levels of GRP mRNA in the DRG than in the SDH. Importantly, GRP knock‐in mice showed expression of GRP in the DRG,^23^ whereas generally used GRP‐promoter Tg mice did not show GRP expression in the DRG. Although they demonstrated that ablation of GRP^+^ neurons in the SDH did not attenuate acute itching, it was still unknown whether activation of GRP^+^ neurons in the CNS directly elicited itching. Despite controversial issue for the source of itch‐related GRP because of the different experimental setting, our present study revealed that central GRP^+^ neurons have an ability to evoke itching in both sexes.

Given that GRP‐Cre (Tg) mice only reflect GRP expression in the CNS and not in sensory neurons and as we previously reported that GRP promoter‐driven expression of tdTomato was observed in the SDH,^15^ Tg mice may be useful for understanding the central roles of GRP^+^ neurons in itch processing. In fact, chemogenetic activation of GRP^+^ neurons significantly elicited scratching behavior associated with neural excitation in the SDH (Figure 3). Based on a previous study demonstrating that GRP^+^ neurons seldom express c‐fos following i.d. administration of chloroquine,^20^ it was hypothesized that c‐fos^+^ cells observed in the SDH might be itch‐responsive GRPR^+^ neurons. Notably, CNO‐induced scratching behavior in GRP‐hM3Dq mice was not affected by RTX‐mediated ablation of the TRPV1^+^ sensory neurons, indicating that activation of GRP^+^ neurons in the SDH could induce central itching sensation (Figure 7C). Although Barry et al. indicated that ablation of spinal GRP^+^ neurons did not affect pruritogens‐mediated acute itching,^23^ the transmission process of itching sensation after primary sensory neurons has not been completely understood. Albisetti et al. also demonstrated that chemogenetic activation of spinal GRP^+^ neurons by injecting adeno‐associated virus into the SDH caused itching‐related biting behaviors in mice,^32^ which is conceptually consistent with our findings. This suggests that spinal GRP^+^ neurons clearly contribute to itch processing. Interestingly, CNO administration markedly enhanced scratching behavior in DCP‐treated GRP‐hM3Dq mice (Figure 7D). Although there might be a possibility of independent mechanisms between CNO‐ and DCP‐elicited itch, it was hypothesized that the itch‐inducible roles of spinal GRP^+^ neurons were amplified under chronic itching conditions, as the expression level of GRP was markedly increased in the SDH under chronic itching conditions, and that chemogenetic inhibition of GRP^+^ neurons attenuated DCP‐elicited itch in our previous report.^15^

As suggested that GRP is released from glutamatergic interneurons in the SDH, different groups of researchers suggested that activation of GRPR^+^ neurons through α‐amino‐3‐hydroxy‐5‐methyl‐4‐isoxazolepropionic acid receptor (AMPAR) by glutamate is a key component of itch processing in the SDH.^33^, ^34^, ^35^ We also recently demonstrated that GRP and glutamate cooperatively activate itch‐responsive GRPR^+^ neurons in the SDH under acute and chronic itching conditions.^15^ Notably, i.d. chloroquine or compound 48/80‐induced firing of spinal GRPR^+^ neurons was attenuated by a combination of GRPR and AMPAR antagonists,^15^ but not by GRPR antagonist alone, supported by behavioral experiments, indicating that the glutamate‐AMPAR system is a fundamental factor in itch processing from the periphery to the SDH under normal conditions. On the other hand, Pagani et al. found that burst firing of spinal GRP^+^ neurons released enough GRP to excite GRPR^+^ neurons, while single action potential of GRP^+^ neurons failed to release sufficient GRP.^36^ Moreover, such phenomena were blocked by GRPR antagonist, but not by AMPAR antagonist, indicating that excessive activation of GRP^+^ neurons in the SDH might have additional potent effects on GRPR^+^ neurons. Given that the expression level of GRP was markedly increased in the SDH under chronic itching conditions, as previously reported,^15^, ^27^, ^37^ functional significance and susceptibility of spinal GRP^+^ neurons can be changed based on pathophysiological conditions. Spinal GRP^+^ neurons may have potent effects on itch processing through GRP and/or glutamate actions under both physiological and pathological conditions.

The central effects of the GRP‐GRPR system on itch processing have been previously demonstrated by both i.t. and i.c.v. administration of bombesin and GRP in rodents.^38^, ^39^ These reports suggest that the GRP‐GRPR system also plays a role in the itching sensation in the trigeminal innervation area, and i.c.v. administration of CNO in the GRP‐hM3Dq mice elicited scratching behavior in the face/head in this study (Figure 6). Similar to the effects seen with i.t. administration, the effects of i.c.v. administration might mainly occur in the face/head area. Interestingly, expression of HA‐hM3Dq was mainly observed in the facial nucleus, but not the spinal trigeminal nucleus (Figure 5). It is generally known that nociceptive information in the trigeminal area is transmitted to the trigeminal nucleus,^40^, ^41^ and expressions of pain or itch‐related neuropeptides (i.e., calcitonin gene‐related peptide and GRP, respectively) were observed in such regions.^42^, ^43^ Moreover, it was reported that GRPR^+^ neurons were abundantly located in the spinal trigeminal nucleus‐caudal part and SDH.^44^ Nevertheless, in GRP‐Cre (Tg) mice, the activity of the GRP promoter in the facial nucleus, including motor neurons, might be greater than that in the trigeminal nucleus. This is supported by a previous report showing that marked expression of GRP‐tdTomato in the facial nucleus and various brain regions, such as the cortex, thalamus, and midbrain.^32^ Since the GRP‐GRPR system has an interesting role in the itch processing in the brain, for example, GRPR^+^ neurons in the suprachiasmatic nucleus mediate contagious itch,^45^ further investigation is needed to understand the role of the supraspinal GRP‐GRPR system in itch processing.

Recently, sex differences in the pathophysiology of several diseases have received attention.^46^, ^47^, ^48^ In pain processing in humans, it was demonstrated that the pain threshold in females was lower than that in males.^49^, ^50^ Moreover, there are considerable differences in the molecular mechanisms of chronic pain, as spinal microglia play a pivotal role in neuropathic pain in males but not in females.^51^, ^52^ Thus, therapeutic intervention for pain in females may often be more difficult than that in males. However, there is no evidence showing the sex difference in itch processing. Because majority of previous reports that investigated itch mechanisms were performed using male mice, it is pivotal to examine whether there is sex‐dependent regulation of itching. Based on our present findings, GRP^+^ neuron‐mediated central itching may occur at a similar degree in both males and females. Given that the GRP‐GRPR system is known to be itch‐dominant, it is reasonable that chemogenetic activation of spinal GRP^+^ neurons did not affect the pain sensitivity to thermal or mechanical stimuli, which is supported by several previous reports.^14^, ^16^, ^32^ Unlike pruritogens or neuropeptide‐mediated acute itching, chronic itching is largely affected by immune systems underlying intractable skin diseases.^5^, ^53^ Because there are sex‐related differences in immune systems,^54^, ^55^, ^56^ there is a possibility that the degree of chronic itching could differ between males and females. Nevertheless, sex‐related differences in neurochemical systems, but not immune systems, regulating itching under normal and pathological conditions are completely unknown. In order to develop effective treatments for intractable itching, it is imperative to understand the sex‐dependent regulation of itch processing.

Collectively, we demonstrated that chemogenetic activation of central GRP^+^ neurons by CNO administration (s.c., i.t., or i.c.v.) in GRP‐hM3Dq mice elicited robust dermatome‐dependent scratching behavior, which was not affected by ablation of the TRPV1^+^ sensory neurons. Furthermore, this was observed to a similar degree even under chronic itching conditions. Notably, there were no significant sex differences in scratching behavior elicited by Gq‐DREADD in spinal GRP^+^ neurons, suggesting that itch‐processing roles of GRP^+^ neurons might be common in both sexes at least under physiological conditions. These novel evidence lines not only contribute largely to understanding the functional roles of GRP^+^ neurons further by addressing the current controversial issue regarding the GRP‐GRPR system in itch processing, but also propose the development of future effective therapeutics for intractable itching.

## DISCLOSURE

The authors declare no conflict of interest.

## AUTHOR CONTRIBUTIONS

Participated in research design: Kiguchi, Fukazawa. Conducted experiments: Kiguchi, Fukazawa, Saika A. Performed data analysis: Kiguchi, Fukazawa, Uta, Saika F., Nakamura, Ko, Kishioka. Wrote or contributed to the writing of the manuscript: Kiguchi, Fukazawa, Ko, Kishioka.

## Supporting information

Figure S1

## Data Availability

The data that support the findings of this study are available from the corresponding author upon reasonable request.
