# Supplementary material for: Chemogenetic activation of central gastrin‐releasing peptide‐expressing neurons elicits itch‐related scratching behavior in male and female mice
Source: Pharmacol Res Perspect. 2021 May 17;9(3):e00790. doi: 10.1002/prp2.790 (PMC8128314; doi:10.1002/prp2.790)
Supplement: Supplementary file 1 — Figure S1 [file PRP2-9-e00790-s001.docx]

*Pharmacology Research & Perspectives*

**Chemogenetic activation of central gastrin-releasing peptide-expressing neurons elicits itch-related scratching behavior in male and female mice**

Norikazu Kiguchi, Yohji Fukazawa, Ayano Saika, Daisuke Uta, Fumihiro Saika, Tomoe Y. Nakamura, Mei-Chuan Ko, Shiroh Kishioka

*
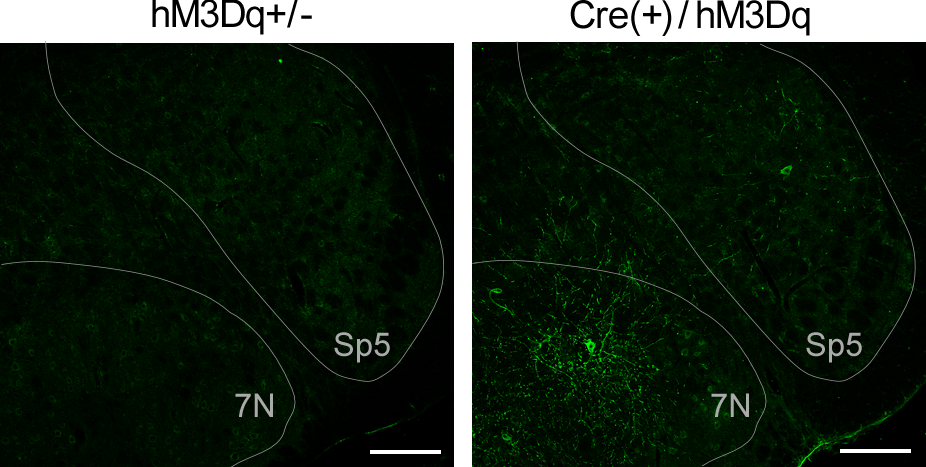
*

**Supplemental figure**

Fig. S1 Expression of gastrin-releasing peptide -Cre-driven Gq- designer receptors exclusively activated by designer drugs system in the brain

The Cre-dependent expression of HA-tagged hM3Dq in the facial nucleus and the spinal trigeminal nucleus oral part of male GRP-hM3Dq mice, but not of hM3Dq heterozygous (control) mice, was visualized using immunohistochemistry (Scale bars = 200 µm).

(GRP, gastrin-releasing peptide; HA, hemagglutinin)
